# How young people experienced COVID‐19 disease containment measures in the Western Cape, South Africa: A qualitative study including the perspectives of young people, their parents, teachers and school counsellors

**DOI:** 10.1111/papt.12374

**Published:** 2021-12-13

**Authors:** Bronwynè J. Coetzee, Hermine Gericke, Suzanne Human, Paul Stallard, Maria Loades

**Affiliations:** ^1^ Department of Psychology Stellenbosch University Stellenbosch South Africa; ^2^ Department for Health University of Bath Bath UK; ^3^ Department of Psychology University of Bath Bath UK

**Keywords:** adolescents, COVID‐19, disease containment measures, LMICs, mental health, pandemic, qualitative, South Africa

## Abstract

**Background:**

Little is known about the potential impact of COVID‐19 disease containment measures on children's mental health and well‐being, particularly in low‐ and middle‐income countries. We sought to explore this amongst young adolescents in South Africa and from the perspectives of multiple key stakeholders.

**Methods:**

We conducted 25 individual semi‐structured telephonic interviews with children (*n* = 7, aged 12–13 years), teachers (*n* = 8), parents/caregivers (*n* = 7) and school counsellors (*n* = 3) from two public primary schools in the Western Cape, South Africa. Interviews were conducted between July and September 2020 and transcribed verbatim. The data were analysed inductively using thematic analysis procures.

**Results:**

We generated three overarching themes: “locked down at home”, “social disconnection” and “back to school.” Children had varying reactions to COVID‐19 and lockdown including excitement, frustration, anxiety, boredom and loneliness. Parents were anxious about teaching, and technology did not consistently provide the necessary support. Children felt disconnected from their peers at home, and at school, reconnecting with friends was obstructed by disease containment measures. All participants were concerned about children completing the academic year successfully and worried excessively about the implications of this year on their future.

**Conclusion:**

Young people and their immediate networks, in a low‐ and middle‐income context, described a variety of negative impacts of disease containment measures emotionally, although there was a wide variety of experiences. Children, parents, teachers and counsellors all wanted resources and support and were concerned about the longer‐term impacts of disease containment measures.

**Practitioner points**

- Children in this study had varying reactions to lockdown and the return to school, and these need to be closely monitored as some will need more support than others.
- Children need clear and careful explanation of the reasons for disease containment measures and how they can protect themselves and others to minimise anxiety.
- Children's social and emotional well‐being needs to be prioritised during lockdown and after, alongside their academic progress.
- Parents need support to create structure and routine and keep children engaged in these, which will help minimise both their own and their children's anxiety.
- Children need to maintain connection with their peers as an important source of support.

## BACKGROUND

**FIGURE 1:**
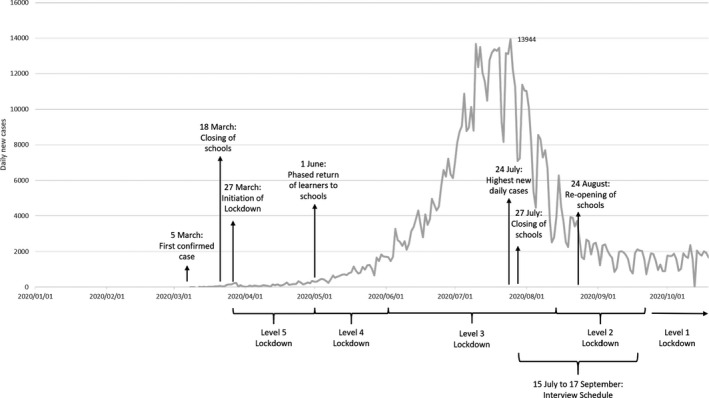
Progression of COVID‐19 in South Africa and indication of various levels of lockdown

The Coronavirus (COVID‐19) pandemic is having a profound effect on all aspects of society, including mental health (Holmes et al., 2020). On the 30th of January 2020, the World Health Organisation (WHO) declared the COVID‐19 outbreak a pandemic and public health emergency. To curb transmission, governments worldwide implemented disease containment measures (DCMs), including hand sanitising, social distancing, home confinement (“lockdown”), quarantine and mandatory mask wearing. In South Africa, a national lockdown commenced on the 27th of March, following the first reported case of COVID‐19 on 5 March 2020 (Mkhize, 2020; see Figure 1 for timeline). During lockdown, South Africans could only leave their homes when it was essential and schools were closed for 10 weeks.

Although DCMs helped to reduce the spread of the infection, there was concern about a number of unintended adverse consequences for children and young people (CYP). Learning was disrupted, and social and emotional support was reduced (Crawley et al., 2020). Access to other services including safeguarding and health care was limited (Golberstein et al., 2020). Children became more sedentary and had fewer opportunities to play (Moore et al., 2020) with home confinement increasing feelings of loneliness, which are associated with anxiety and depression (Loades et al., 2020).

There is emerging quantitative evidence from across the world that COVID‐19 is impacting on the mental health of CYP, particularly relating to depression and anxiety (Nearchou et al., 2020). However, these quantitative studies are limited by their focus on average experience on standardised measures, most of which were developed prepandemic, rather than exploring the variety and complexity of lived experiences of this unprecedented and highly unusual circumstance. Most quantitative studies in the pandemic context have also tended to rely on online data collection (Pierce et al., 2020), which is unlikely to be feasible in a resource‐poor context.

Qualitative studies of the experience of DCMs in CYP, particularly in high‐income countries, have highlighted the diversity of experiences, both adverse and positive. In the United Kingdom, adolescents (aged 16–19 years), recruited via social media, described their experiences of lockdown in an anonymous online survey (Demkowicz et al., 2020). They reported that lockdown was a time of change, loss and uncertainty, heightened emotionality and frustration with the government and the media. Lockdown was also an opportunity for growth and development, with many adolescents trying to stay positive and hopeful, valuing self‐care and highlighting the importance of having a sense of togetherness (Demkowicz et al., 2020). In another UK‐based qualitative study, researchers conducted focus groups with parents (30–50 years) and CYP (14–15 years; Sajid & Saleem, 2020). CYP reported difficulty in home schooling and the loss of routine due to school closures. Although CYP assisted parents in household tasks, they wished to spend more quality time with their parents. CYP also expressed worries regarding their future education and job prospects and said that they missed social interactions with friends. Both parents and CYP expressed the importance of routine in creating a sense of normalcy. However, parents reported worrying about their children's health and mental health, children spending too much time on digital devices and their education (Sajid & Saleem, 2020).

Research into the lived experiences of CYP, and the potential effects of COVID‐19 and DCMs on their mental health, especially in low‐ and middle‐income countries (LMICs), such as South Africa, remain lacking. Not only do CYP make up 35%–50% of the population in LMICs (Patel et al., 2008), but it is also likely that the prevalence of mental health problems among CYP in LMICs are even higher than the worldwide estimate of 13.4% (Polanczyk et al., 2015), because children in these countries are exposed to multiple risk factors such as violence, child maltreatment, living in households affected by HIV/AIDS and poverty (Flisher et al., 2012; Kieling et al., 2011; Lu et al., 2018; Patel et al., 2007). In South Africa, where large proportions of the population live in uncertain circumstances, DCMs such as home confinement forced millions of people to spend lengthy periods of time together in close proximity to one another and with limited resources (Coetzee & Kagee, 2020). Furthermore, the mental health needs of CYP are not being adequately addressed in LMICs due to a lack of availability of services, a lack of access to services and limited intervention research in these contexts (Bradshaw et al., 2021). Thus, even before the COVID‐19 pandemic, many CYP living in a LMIC, such as South Africa, were already especially vulnerable to developing mental health problems (Cortina et al., 2013; Das‐Munshi et al., 2016; Kieling et al., 2011; Lund et al., 2011; Patel et al., 2007; Perold, 2001). In South Africa, there are no national estimates of the prevalence rates of mental health problems in CYP. However, data from the Western Cape province (where this study took place) indicate that 17% of children living in the province have a clinical disorder (Kleintjes et al., 2006). In this study, the authors show that for children and adolescents in the province, generalised anxiety disorder (11%), post‐traumatic stress disorder (8%) and major depressive disorder (8%) were the most common mental disorders. These findings coincide with estimates of anxiety symptoms amongst CYP (7–13 years) in the Western Cape, reporting prevalence rates of 22%–25.6% (Muris et al., 2002).

We undertook a qualitative study to explore the ways in which COVID‐19 and DCMs in South Africa influenced the lives of a sample of children attending primary schools in the Western Cape, South Africa, shortly after the initial lockdown in South Africa in 2020. We also undertook to interview their parents/primary caregivers/legal guardians (henceforth referred to as parents), teachers and school mental health counsellors to elicit their perspectives on the influence COVID‐19 and DCMs had on children's lives during this time. As such, our research question was the following: How did a sample of primary school‐aged children in South Africa experience national lockdown and school closures in 2020? Our subquestions were the following: (1) What were children's experiences of the national lockdown and school closures and how did this differ from how things were before? (2) What were children's parents, teachers and school counsellors’ thoughts on the ways in which the national lockdown and school closures impacted on children's lives, with particular reference to the impact on their emotional health and well‐being?

## METHODS

### Research context

This study forms part of a larger study, aimed at developing and assessing the acceptability and feasibility of a Cognitive Behavioural Therapy (CBT)‐based psychoeducational intervention to support the psychological well‐being of children in primary schools in the Western Cape, South Africa (Trial ID: PACTR202004803366609). This qualitative substudy took place in collaboration with two urban, public primary schools in the Western Cape province of South Africa and a non‐government organisation (NGO) that provides children in these schools with psychosocial support services. The two schools were randomly chosen (using computer randomisation) from a list of schools within which the NGO operates. As can be seen from Figure 1, at the time of our interviews (in July–September 2020), our participants had already experienced the initial lockdown period in South Africa (which commenced on 27 March 2020), as well as two school closures; the first following the initial lockdown and the second on 27 July 2020. The first school closure period was just over 2 months long (18 March 2020 to 1 June 2020), and the second school closure period was also approximately 1 month long (27 July 2020 to 24 August 2020). In between these school closures, the Department of Basic Education recommended a phased reopening of schools, where learners would attend classes on alternate days to honour DCMs while teachers returned full time.

### Participant identification and recruitment

**FIGURE 2:**
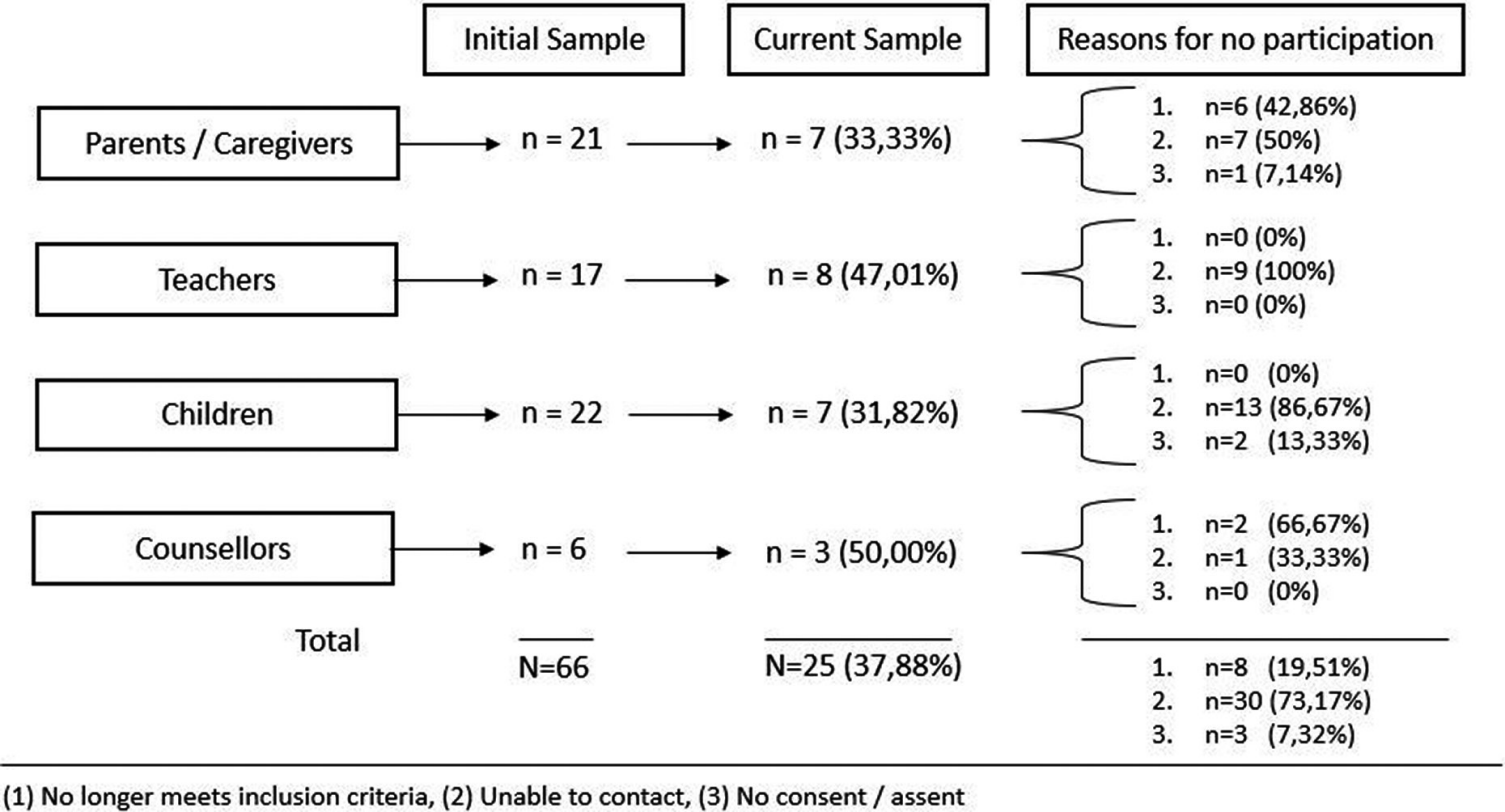
Attrition of participants from initial interviews in 2019 to follow‐up interviews in 2020

As part of our larger study and approximately 1 year prior to the current study, we used convenience sampling to recruit participants from the following key stakeholder groups at the selected schools: CYP (male and female) in grades 5–7 (approximately 10–15 years of age); parents of said CYP; teachers of said CYP; and school mental health counsellors (NGO staff). In this substudy, we received ethics permission to invite participants who took part in the initial interviews, for a follow‐up interview, telephonically. We used existing contact details of participants who took part in the larger study to invite participants for this study. As can be seen in Figure 2, we had an initial sample of 66 participants from the first study who we could invite and include in this study. Further, as can be seen from Figure 2, we were able to include 25 of the participants from the initial sample, and we provide reasons for non‐participation of the other participants. For example, eight participants no longer met inclusion criteria, 30 participants were no longer contactable and three participants did not provide consent/assent. In the case of participants who no longer met inclusion criteria, there were six parents whose children had left primary school and were now in high school and two school counsellors who no longer worked at these schools. We were unable to include any new participants in this study, as at the time of our interviews schools were either closed or had restricted access in that no persons from the outside could gain access to the schools. Further, our institutional ethics committee suspended in‐person data collection during this time.

### Data collection

Given the telephonic nature of the interviews, we obtained verbal consent from parents, teachers and school mental health counsellors. Children's parents provided verbal consent for them to participate, following which children themselves provided verbal assent. We told participants that we have since used the information that they provided to us before to develop a programme, which we are calling 4 Steps To My Future. Participants completed a short demographic questionnaire before the interview began which asked questions about their gender, age and first language. Following the completion of the demographic questionnaire, we conducted an interview with each participant. The interviews were semi‐structured in nature, which allowed for flexibility in the questions asked and was appropriate given the range of participants we spoke to. The interviews were guided by an interview schedule developed for each of the participant groups. Participants were interviewed by the same members of the research group [SH; HG] who interviewed them before. Interviews were conducted from July to September 2020.

In the interviews with CYP, we asked participants, initially, how they kept busy during the school closures. We also asked them if they experienced positive things during the national lockdown and school closures and how this lockdown differed from their usual school holidays. We also read children a short case vignette (available on request from corresponding author) about the coronavirus and school closures and then asked them about their own thoughts and feelings on these matters. We asked CYP in what way things differed from before. Finally, we also asked CYP what makes them feel happy, sad, angry and scared (anxious/nervous) as well as what they think and what they do when they experience these feelings.

In the interviews with parents, teachers and school counsellors, we asked participants about the challenges children faced during and after the national lockdown and school closures and ways in which they coped or managed with these challenges. We also asked these participants whether they thought the pandemic and national lockdown had affected the emotional health and well‐being of children and ways in which difficulties with emotional health and well‐being were dealt with. We also asked participants about the challenges children faced (if any) since the reopening of schools.

### Data analysis

Interviews were audio recorded and transcribed verbatim, and Afrikaans interviews were translated into English. All transcriptions were anonymised and checked for accuracy by another member of the research team. We used ATLAS.ti v8 to assist with thematic analysis of the data (Braun et al., 2019). The data were coded inductively by SH and HG, who consulted frequently with one another and with BC and ML to check the consistency of the codes developed. The data for each group of participants were first coded separately, following which the data were combined and themes and subthemes identified by coders were extracted and compared across the four groups. Iterations of the synthesis of the results were reviewed by all the authors. We ensured rigour and trustworthiness by consulting frequently as a team during the various phases of analysis for consensus on coding and theme development.

### Ethics

This study received ethics approval from Stellenbosch University’s Research Ethics Committee (project number: 9183). Data for this study are available on reasonable request from the authors.

## RESULTS

### Participant characteristics

We conducted 25 (37.88% of original sample) follow‐up telephonic interviews in the COVID‐19 context. Figure 2 shows reasons for attrition.

The participants were seven parents, eight teachers, seven CYP, and three counsellors. Interviews lasted on average for 24.88 min (ranging from 9.85 to 45.57 min, *SD* = 9.65). Participant characteristics are reported in Table 1.

**TABLE 1:** Demographic information of participants (*N* = 25)

Demographic characteristic	Stakeholders			
	Children (*n* = 7)	Parents (*n* = 7)	Teachers (*n* = 8)	Counsellors (*n* = 3)
Age in years				
Mean (standard deviation)	12.43 (0.54)	47 (4.1)	41.38 (11.34)	29.33 (1.53)
Gender				
Female	6 (85.7%)	7 (100%)	4 (50%)	3 (100%)
Male	1 (14.3%)	—	4 (50%)	—
First language				
Afrikaans	5 (71.4%)	6 (85.7%)	7 (87.5%)	3 (100%)
English	2 (28.6%)	1 (14.3%)	1 (12.5%)	—
Current grade at school				
Grade 6	4 (57.1%)	—	—	—
Grade 7	3 (42.9%)	—	—	—
Relationship to child of interest				
Biological parent	—	4 (57.1%)	—	—
Guardian/caregiver	—	3 (42.9%)	—	—
Teaching experience (years)				
Mean (standard deviation)	—	—	13.94 (12.15)	—
Counselling experience at school				
>24 months	—	—	—	3 (100%)

### Themes resulting from data analysis

We generated 3 themes and 11 subthemes across the four participant groups (see Table 2). Each theme is described below with illustrative anonymised quotes. We did not find any themes that were entirely unique to any of our four participant groups. As such, all participants reported similar accounts of children experiences but placed varying degrees of emphasis on the various challenges they faced.

**TABLE 2:** Themes and subthemes identified from thematic analysis

Themes	Subthemes
Locked down at home	Initial excitement to frustration
	What it meant to be in lockdown
	Balancing home, work and school
	Managing new technologies
Social disconnection	Limited social interaction
	Reconnecting with peers
Back to school	Initial unease
	Trying to cope with the new normal
	Concerns relating to the virus
	Other worries are more important
	Academic concerns

### Locked down at home

This theme describes the lived experiences of CYP while under home confinement or “lockdown” and consists of four subthemes: “Initial excitement to frustration,” “What it meant to be in lockdown,” “Balancing home, work and school” and “Managing new technologies.”

#### Initial excitement to frustration

Parents and CYP described how, in the early days of lockdown, children were excited and happy about the prospect of being home, having more time with their parents which allowed for closer family bonding, having a break from school and participating in fun, family‐orientated activities. One child said:

we had a lot of fun together with my family. We watched television, we ate sweets and so on.

One counsellor said that some children were probably better off at home, because their challenges, such as being bullied, are at school:

during the lockdown I also knew a few of my clients are actually better off. I could know with certainty that that child is actually more okay because his challenges lie at school. He's been bullied badly or there are terrible things at school and then I actually had so much reassurance to know that he is where he wants to be, at home with mommy and daddy where it's safe.

Parents described how, as lockdown continued, children's initial excitement about being home faded and was replaced by increasing levels of irritation and frustration. It also became more difficult for parents to convince their children to adhere to lockdown regulations, especially when other children were out and about in the neighbourhood.

Some participants, including children themselves, described how some children felt sad about having to stay home all of the time and concerned about the possibility of illness and death. Some were worried and nervous about either themselves or their parents becoming ill, which seemed to be exacerbated by the possibility of spreading the virus. One child said:

I was scared because you have to always stay at home and not play outside because you can affect people if you have the virus.

Although some children did have feelings of sadness and anxiety relating to COVID‐19, according to parents, teachers and counsellors, other children seemed to not grasp the seriousness of the situation. These children seemed to view lockdown as an extended holiday and went on doing what they would usually do during school holidays, like playing outside with their friends. One teacher stated:

I don't know if our children are taking this pandemic seriously. If I‐ when you're outside if you drive in the road on your way home or in the morning right, then you see children playing together outside of the home without masks. They touch each other, play tag, play in the park together, so I think that they, if they want to get outside of the home they went outside the home. That’s how they handled it like it didn't‐ this pandemic didn't cage them in, understand? They just didn't come to school, but I think they just carried on as normal.

#### What it meant to be in lockdown

In contrast to those children for whom home was a safe place during lockdown, parents, teachers and counsellors reported that, for some children, being locked down at home potentially meant being subjected to adversity, such as lack of adequate nutrition, domestic violence and/or parents who abuse substances. For these children, school may be a safe haven where they feel loved. One teacher stated:

… remember our children have different problems at home. They have parents who are alcoholics, drugs, abuse, […]. They sit in those houses the whole day and‐ poverty where they don't have food. It affects them emotionally, their emotional wellbeing, because they get food here at school they get extra attention and love here at school where it doesn't really happen‐ [...] so it it has a negative effect on their emotional well‐ wellbeing.

For some children, home confinement could also have meant retraumatisation. One girl spoke about seeing a local man who had abused her previously more often during lockdown. This child also disclosed having considerable emotional difficulties during this difficult period.

#### Balancing home, work and school

Parents reported that their children struggled without their routine and the structure created by attending school, and children talked about becoming bored at home especially without being able to see their friends. One of the parents said:

… their habit has always been … out of school do homework, the work that they have to do, then they are outside. That is what they regularly did … that was their usual routine that they knew … and if a child is in that routine it's very difficult … to get him to … adapt to a to a different routine … where he must know the whole time … ‘I have to stay at home, I have to stay at home I'm not allowed outside I shouldn't be outside’.

One of the children said:

Interviewer: “how was it for you when you weren't able to go to school?”

Child: “it was kind of boring because you didn't actually see your friends”

Parents reported that their children struggled to stay busy and stimulated, especially when the family had limited resources. One parent stated:

we don't always have the facilities to keep them inside that they can, how can I say, to keep them busy at home that they don't become so bored. We try to do what we can. Man, I'm going to be honest by saying the‐ our wellbeing‐ like financially and so on is not so strong.

Parents reported that their children found it difficult to stay motivated and engaged in their schoolwork. Parents, themselves, also reported feeling ill equipped to manage their children's learning needs. One of the parents said:

the teacher says do this with the child and then‐ and we do it according to what we understand but I can‐ I don't know if the teacher does it differently. But it's my way, so now I say, if she goes back to school next year or whenever, all of that work will need to be repeated because I don't know if my methods were effective.

One of the children voiced concern about keeping up with schoolwork while at home: “I felt that how are we going to work now and how are the teachers going to explain to us and if we don't understand what are we going to do then?” These worries, combined with the academic pressure that parents placed on CYP to continue to do well academically while being at home, were perceived to fuel children's own anxieties about their academic future.

#### Managing new technologies

Teachers reported that some children had difficulty accessing their school work and keeping updated as children and their families had variable access to devices such as smartphones or computers. One teacher stated:

so I learned okay that's how‐ the things are done. These things you can't do with my type of community, you know? Certain children's parents you can‐ those who have technology because it's the contact‐ the distance teaching doesn't work if you don't have money, if you don't have the technology. It affected me a lot to accept you can't see the children.

School counsellors were concerned about children's privacy and anonymity during remotely delivered therapy sessions, as many children live in overcrowded homes where a private space, to speak over the phone, is unattainable. Counsellors also reported the difficulty of providing adequate therapy during these calls due to children often feeling uncomfortable, difficulty building rapport and lack of confidence on the part of the counsellors themselves. Restricted therapy led to counsellors providing mental health “first‐aid,” that is, short check‐ups to ensure the well‐being of their clients.

### Social disconnection

This theme describes the experiences of CYP regarding their social interaction with their peers during “lockdown,” and upon returning to school. The following subthemes were identified: “Limited social interaction” and “Re‐connecting with peers.”

#### Limited social interaction

During lockdown, children described feeling frustrated about having to stay home and missing their friends, teachers and school. One child said:

… when the president said that we are going into lockdown I was angry with the president because I couldn't go to school to see my school friends.

Children's feelings of frustration were often exacerbated by observing other children engaging socially despite the DCMs. Seeing other children engaging socially had children question the validity of the DCMs and their parents’ expectation for them not to interact with their friends in‐person. One parent said:

the children are, like I say, it's not‐ the parents aren't very strict with the children. Some of their friends are playing outside now automatically our children also want to be outside and it becomes very stressful. Like I said, they can't understand why they can't go outside because the other children are then playing outside.

Participants reported that technology was instrumental to connecting learners with their peers which lessened their feelings of social isolation and helped them cope on a day‐to‐day basis. One of the children said:

I tried to stay in contact with my friends as much as possible and we organised things that we could do when COVID isn't there anymore and planned many other little things.

#### Reconnecting with peers

Because of the social isolation during lockdown, children were excited to return to school to see their friends and teachers again. Although parents tried to manage children's expectations about social interaction at school, given the DCMs, nonetheless, children were disappointed. Children expressed frustration with not being able to go to school on the same days as their friends (due to alternate school schedules to allow for social distancing) and not being allowed to engage physically when playing, in class and showing affection. One of the children said:

we are in two groups the blue group and the yellow group.” Interviewer: “okay and then you take turns to go to school?” Participant: “yes my best friend is not with me at the school.

### Back to school

This theme reports on the experiences of children returning to school in phases, following the “lockdown.” The following subthemes emerged from the data: “Initial unease,” “Trying to cope with the new normal,” “Concerns relating to the virus,” “Other worries are more important” and “Academic concerns.”

#### Initial unease

Teachers reported that in the first few days of returning to school, children were unusually quiet, disengaged and seemed anxious and uncertain about how to act and meet the expectations regarding DCMs. However, over time, these initial reactions seemed to subside. One of the teachers wondered if children's initial unease and behaviour changes when returning to school could be attributed to fear about death and the uncertainty of the virus, and others ascribed it to academic pressure and uncertainty of the schedule of the remaining school year. A teacher said:

Teacher: “at the moment there is more obedience, yes some of them who are a bit noisy, don't listen a bit, I would say are a bit naughty but in general there is very good discipline amongst the children but it's because of fear so and they just do it yes”

Interviewer: “and what do you [sir] think they are afraid of?”

Teacher: “look like I think‐ it can be dying, it's a new thing that no one can give answers to.”

Teachers and counsellors also expressed that children's feelings of anxiety and uncertainty were often compounded by the fears of teachers and parents. Although teachers noticed this change in behaviour with the reopening of schools, children did not remark on their initial feelings and behaviours and how this might have changed over time. However, children talked about their dislike of the DCMs that they are expected to follow. One of the children said:

Interviewer: “how was it when you did go back to school?”

Child: “when we were at school you‐ we had to sit a meter away from each other [...] and we couldn't actually play with each other because of the Coronavirus [...] and every time we go out somewhere then we come back then we sani‐ even when we went to the bathroom sanitize after eating sanitize every time.”

#### Trying to cope with the new normal

Parents stated that they prepared their children for the return to school by carefully explaining that the government decided to follow a phased return to school strategy and to implement DCMs at school to limit the spread of the virus. However, parents reported that children often found these explanations incomprehensible, making it difficult to contain or soothe children's concerns and uncertainties. One parent said:

I think they understand the crisis surrounding Corona and stuff but what you see ‘if Corona is so dangerous that no one may go to school why can [name of sibling] go to school then’ is almost like ‘I don't understand it [name of sibling] may go so I can also then go’ so it cost a bit of time and effort to get that message across to them that we are going to phase it in.

Other attempts to mitigate children's concerns over risk of exposure included parents creating strategies and routines at home when children returned from school, such as handwashing stations at entrances and immediately removing and washing school clothes. A parent said:

When she comes home from school here's a thing that we have taught everyone is maybe‐ when you come in by the door is to wash hands and there's a ready‐made bowl with jik ((bleach)) water to wash their hands in.

#### Concerns relating to the virus

There was considerable variability in children's anxieties about returning to school amidst the pandemic. Parents, teachers and school counsellors reported that children were concerned about being exposed to the virus and becoming ill. One of the parents said:

they were a bit worried about getting ill, they were worried about washing hands and wearing masks and why all of these things.

Furthermore, parents, teachers and school counsellors reported that children expressed concern over the possibility of being infected with the virus, infecting others and loved ones becoming ill and dying. Similarly, children themselves also expressed fear regarding exposure to the virus. One of the children said:

Interviewer: “okay and [name of participant] what makes you feel anxious or scared or nervous?

Child: “uhm I'm afraid I take off my mask and now it's at a place where there is a lot of people and then and then I'm afraid I get COVID‐19 miss [...] I will perhaps have to go into quarantine miss and then I can never come out again” [...]

Interviewer: “mm what do you feel a bit nervous about?”

Child: “uhm maybe I go out without a mask and then I infect someone miss.”

These feelings of concern were often exacerbated by surrounding people not adhering to DCMs and positive cases at school.

#### Other worries are more important

Upon returning to school, teachers and school counsellors noted that there were not many referrals to the school mental health counsellors for COVID‐19‐related anxieties and that children's worries and referrals to the school mental health counsellors were often related to other situations.

I haven't seen really serious anxiety amongst the children that, you know, is so bad that I had to refer to [name of collaborating NGO]. She has I would say her regulars (laughs) but I don't think there has, in the meantime, been additional children specifically due to the COVID‐19 pandemic. I think it was other stuff Teacher

Children shared concerns related to being bullied or teased, seeing other children getting bullied, parental divorce, exposure to community violence or hearing news reports of stabbings or kidnappings. One child said:

Interviewer: “What makes you feel sad?”

Child: “When someone bullies me or hits me.”

Teachers felt that children were more concerned with their community and home problems than their exposure to and the challenges of the virus itself, stating “it's just another thing so and I don't think it's their biggest worry.” A counsellor reported that children and parents were focused on surviving lockdown and its challenges, thus placing a hold on dealing with mental health issues unrelated to COVID‐19. The counsellor said:

everyone is focusing on academics and to just survive, like what the COVID means and to make it through the lockdown and I think now that we have returned to school, we can almost unpause again and get the other types of references ((referrals)) again.

#### Academic concerns

Children, teachers and parents expressed concern regarding the shortened academic year and how this would impact the children's academic future. Some parents were so worried about their children falling behind with their schoolwork that they decided to send them back to school, despite their children having pre‐existing medical conditions that may exacerbate their risk of becoming ill. One of the teacher's said:

I also have one learner in my class and this learner just decided he has asthma. He should actually be at home but he decided, no, he can't because he's falling behind, it's not the same. So his parents contacted me and asked whether he could return to school, so then we said okay as long as you know there's a risk associated with it and you're comfortable with the risk.

However, despite anxiety over academic progression, teachers noted that many children struggled with motivation and concentration during lockdown. Consequently, much of what is left of the shortened academic year is being spent on revisiting concepts covered during lockdown. A teacher said:

but the problem is just that when the child returns I have to repeat everything, hey? Which is also a good thing but it's also a time‐consuming task and it means that I won't be able to get to everything.

Teachers also reported that with the continual closing and reopening of schools, children seemed confused and uncertain, leading to anxiety and fear amongst them. Furthermore, the confusion and uncertainty might also have led to a distrust in the education system. One of the teachers said:

I think it confused them a lot. I think there was always a certainty, you know, through‐ this is the holiday this is how tomorrow's going to look like, you know? You go to school and now they don't have the certainty anymore, it was taken away from them, so I think for them it's a little stressful to not know anymore‐ and it can cause anxiety to not know what's happening tomorrow and the education department's playing around with dates also caused them to lose faith in the system. They don't know what to expect.

Although children mentioned their concerns about the impact of the pandemic on their academic future, few commented on a lack of motivation and concentration, how the school has been coping with teaching or their distrust in the education system.

## DISCUSSION

Children in an LMIC such as South Africa had variable and changing experiences of lockdown, ranging from excitement to frustration and boredom. For some, positives of home confinement included spending more time with their parents and escape from difficult situations at school like bullying and teasing. For others, lockdown was challenging and potentially traumatic. Parents especially felt ill equipped to support their children's learning needs and keeping them engaged in their schoolwork, which compounded anxieties about academic progress. Limited and inconsistent access to technology added to the pressures of doing schoolwork online while being locked down at home. During lockdown, children felt disconnected from their peers. On returning to school, reconnecting with peers was limited by ongoing DCMs. Children needed to initially adjust to returning to school and experienced many uncertainties about the new school arrangements and classroom environment. Children worried about death and the unpredictability of the future and about catching up with schoolwork. They also worried about how DCMs would be consistently applied in practice. Clear explanations from parents helped the adjustment process. Although concerns about the virus were present among children, they quickly became more concerned by worries about bullying and teasing, parental divorce and exposure to community violence as these concerns remain more pressing issues in their lives.

Children in our study experienced a range of psychological, emotional and social reactions over the course of lockdown and on the return to school, both within and between individuals. These reactions included feelings of frustration, irritation and boredom at being home without peers, uncertainty and worry about the future, fear of infection and concerns about death. Similar findings have been reported elsewhere in the COVID‐19 pandemic context (Abdulah et al., 2020; Demkowicz et al., 2020; Jiao et al., 2020; Sajid & Saleem, 2020), although (at the time of this writing) ours is the first study to explore this in South Africa. These findings are also consistent with reactions to quarantine and isolation experiences during disease outbreaks like SARS (Hawryluck et al., 2004; Li et al., 2004) and Ebola (Etard et al., 2017; Hugo et al., 2015).

In many ways, the reactions to COVID‐19 DCMs echo what is known about children's reactions to major events and disasters (Silverman & La Greca, 2002). Valent (2000) describes five phases of reactions to disasters: pre‐impact (the periods before the disaster), impact (when the event happens), recoil (the period immediately after the event), post‐impact (the days and weeks after the event) and, lastly, recovery and reconstruction (the months or years after the event; Valent, 2000). However, much of the available literature on the psychological impact of disasters on children capture the recovery and reconstruction period and are tied to the growing body of evidence on post‐traumatic stress disorder (PTSD) following a traumatic or disastrous event (Silverman & La Greca, 2002). Similarly, studies of the mental health impacts of quarantine in children have tended to be during this recovery and reconstruction period (Sprang & Silman, 2013). Very little literature is available on the other phases, like the impact phase, which is most consistent with the period we are reporting on herewith. Our findings indicate that this impact phase may be characterised by boredom and frustration, academic concerns and concern about the future, consistent with other reports in this context (Abdulah et al., 2020; Demkowicz et al., 2020; Jiao et al., 2020; Sajid & Saleem, 2020), as well as loneliness (Loades et al., 2020).

We found that children's anxieties and concerns were exacerbated by those of their parents. We know that parental stress and mental health issues predict mental health problems in CYP (Deater‐Deckard & Panneton, 2017) and that this is likely to be exacerbated during a disaster or pandemic, especially amongst vulnerable children living in LMICs (Fouché et al., 2020; Simba et al., 2020). Further, adults who experience immense stress are also likely to be less observant and aware of the mental health challenges their children face and may falsely assume their children are doing well and are resilient (Silverman & La Greca, 2002). In our study, parents experienced considerable stress and anxiety about acting as teachers to their children during lockdown and keeping their children safe, engaged and entertained. Similarly, teachers were concerned about children's access to and engagement with learning materials during lockdown. As such, children were not only dealing with their own concerns but may have found the adults in their direct networks to be less available than normal and in some instances, their concerns may have been exacerbated by the worries and anxieties of their parents and teachers.

Although many children may be sufficiently resilient to manage the challenges posed by the DCMs, some will likely need additional support, particularly in a context like South Africa where mental health problems were already common even prepandemic (Cortina et al., 2013; Das‐Munshi et al., 2016; Perold, 2001). In our study, some participants assumed that children were not taking the pandemic seriously and saw it more as an extended holiday. These assumptions may be problematic in that children's reactions to adverse events are likely to differ considerably, which could mean that those children who need support could be overlooked (Silverman & La Greca, 2002).

It was encouraging to learn of coping mechanisms that children, their parents, teachers and counsellors had in place to manage this difficult time, demonstrating their ability to be resilient in the face of this global challenge. Consistent with the available literature, we found that access to social support, routine and structure was important but not always possible (Golberstein et al., 2020). Technology was often unreliable and also mostly inaccessible to children, who required it for online learning and to remain connected to their peers and for social support outside of the immediate family. Indeed, social media and other digital technologies could have the potential to mitigate the severity of the effects of social deprivation (Orben et al., 2020) but only if consistently available and reliable. Although many CYP in other contexts may have been able to turn to technological solutions, our work highlights that there are those for whom their home context and access to resources may make that difficult or even impossible.

### Strengths and limitations

Our findings complement the findings of recent quantitative studies on the overall impact of the pandemic and DCMs on the mental health and well‐being of CYP, by providing an in‐depth and qualitative understanding of CYP's variable lived experiences of the COVID‐19 pandemic.

Telephonic recruitment and interviewing allowed for rich data collection and a wider catchment, reaching people who otherwise might have been missed by relying on online platforms to recruit and collect data. We were only able to conduct follow‐up interviews with a little more than a third of the initial participants and those are likely to have been the participants most able to access a telephone, so we still may not have captured the experiences of those without access to this resource. Most interviews were conducted in Afrikaans and translated into English; therefore, some of the nuances of language may have been lost in the process of translation.

Although the data that we collected showed that some factors predominantly related to the pandemic (such as being scared of becoming ill or dying and not going to school on the same day as your friends) could have contributed to mental health problems amongst CYP, our data also showed that other, ongoing and pre‐existing factors, unrelated but exacerbated by the pandemic (such as not having access to technological resources; community violence; child abuse; domestic violence; substance abuse; and parental divorce), were perhaps more likely to contribute to mental health difficulties among CYP. For the data that we have collected qualitatively, it is complex and nearly impossible to identify all factors related to mental health and well‐being that are strictly related to the pandemic. Indeed, what we do attempt to articulate is that much of the existing mental health issues in our context was likely exacerbated by the pandemic (Daniels, 2021).

### Clinical and research implications in a LMIC such as South Africa

Our work has highlighted the support needs of children and their parents and their ability to cope with the challenges of COVID‐19 DCMs and the emotional impacts of this. Providing clear and consistent information would help to reduce anxiety by addressing uncertainty. Understanding the emotional and psychological experiences at each phase of the pandemic could help to plan appropriate intervention strategies in the short term (such as instrumental and emotional support for children, teachers and parents) and to understand the cognitive, developmental and mental health impacts of COVID‐19 in the longer term. Even across the disaster literature, virtually nothing is known about why some children might show different trajectories of responses and how those patterns might vary with specific disaster characteristics (Silverman & La Greca, 2002). Understanding this is crucial in making the distinction between normal reactions and experiences from those that might interfere with children's day‐to‐day functioning and thus may be pathological (Silverman & La Greca, 2002).

Currently, many countries globally, including South Africa, are experiencing a second or third wave of COVID‐19. In the absence of suitable parental and mental health support, as well as access to social and protective services, children from these communities are likely to continue to experience the economic hardships associated with COVID‐19, as well as the many adverse home (exposure to domestic and interpersonal violence and child and substance abuse) and community (violence, gangsterism and shootings) experiences that are typical of daily life in this context. Those working in these contexts need to be aware that, for some children, life during lockdown has been fraught with danger and potential trauma, in the absence of the usual identification and safeguarding structures within schools.

Future mental health interventions that are universal and underpinned by psychological theory and are focused on resilience and skills building may be important for prevention and/or early intervention in the lives of CYP. A recent systematic review found that CBT‐based interventions have showed some promise in LMIC contexts (Bradshaw et al., 2021), although they have not yet been extensively trialled in South Africa specifically. The lack of ready access to the internet also has implications for the future implementation of mental health interventions, as there are many barriers to accessing digital resources and online mental health interventions in this context. Delivering mental health interventions within school and community settings wherever possible may be the best solution in the current context.

## CONCLUSION

Our findings are amongst the first qualitative findings (Abdulah et al., 2020) on the influence of COVID‐19 DCMs on children's mental health and well‐being and add to a growing body of literature on children's reactions to the COVID‐19 crisis and the psychological, emotional and social impact of school closures and phased reopenings, especially in South Africa. Although reactions, emotional and behavioural responses varied, we found that parental and teacher stressors, as well as ongoing exposure to adverse experiences, compounded children's anxieties about their future. Our findings demonstrate that there is much to understand about the trajectory of responses to natural disasters and pandemics during their different phases, in order to distinguish between understandable/expected (“normal”) and pathological reactions. Understanding more about the unique challenges faced in each phase of a pandemic or disaster will inform interventions to support children, their families and their schools in future. Future research focusing on prevention as intervention that prioritises resilience through skills building may be important and protective against the onset of future mental health problems.

## CONFLICTS OF INTEREST

The author(s) have declared that they have no competing or potential conflicts of interest.

## AUTHOR CONTRIBUTION

**Bronwynè J. Coetzee:** Conceptualization (equal); Formal analysis (equal); Funding acquisition (equal); Methodology (equal); Writing – original draft (equal); Writing – review & editing (equal). **Hermine Gericke:** Conceptualization (equal); Formal analysis (equal); Investigation (equal); Methodology (equal); Project administration (equal); Visualization (equal); Writing – review & editing (equal). **Suzanne Human:** Conceptualization (equal); Formal analysis (equal); Funding acquisition (equal); Investigation (equal); Methodology (equal); Project administration (equal); Visualization (equal); Writing – review & editing (equal). **Paul Stallard:** Conceptualization (equal); Formal analysis (equal); Funding acquisition (equal); Writing – review & editing (equal). **Maria Loades:** Conceptualization (equal); Formal analysis (equal); Funding acquisition (equal); Methodology (equal); Writing – review & editing (equal).

## AUTHOR PERSONAL STATEMENT

Dr. Loades is funded by the National Institute for Health Research (NIHR Doctoral Research Fellowship, DRF‐2016‐09‐021). This report is independent research. The views expressed in this publication are those of the author(s) and not necessarily those of the NHS, NIHR or the Department of Health and Social Care.

## Data Availability

These data are available on reasonable request from the corresponding author.

## References

[papt12374-bib-0001] Abdulah, D. M. , Abdulla, B. M. O. , & Liamputtong, P. (2020). Psychological response of children to home confinement during COVID‐19: A qualitative arts‐based research. International Journal of Social Psychiatry, 002076402097243. https://journals.sagepub.com/doi/full/10.1177/0020764020972439 10.1177/0020764020972439 33183155

[papt12374-bib-0002] Braun, V. , Clarke, V. , Hayfield, N. , & Terry, G. (2019). Handbook of research methods in health and social sciences. In P. Liamputtong (Ed.), Handbook of research methods in health and social sciences (pp. 843–860). Springer Nature Singapore Pte Ltd.

[papt12374-bib-0003] Bradshaw, M. , Gericke, H. , Coetzee, B. J. , Stallard, P. , Human, S. , & Loades, M. (2021). Universal school‐based mental health programmes in low‐and middle‐income countries: A systematic review and narrative synthesis. Preventive Medicine, 143, 106317. 10.1016/j.ypmed.2020.106317 33159922

[papt12374-bib-0004] Coetzee, B. , & Kagee, A. (2020). Structural barriers to adhering to health behaviours in the context of the COVID‐19 crisis: Considerations for low‐ and middle‐income countries. Global Public Health, 15(8), 1–10. 10.1080/17441692.2020.1779331 32524893

[papt12374-bib-0005] Cortina, M. A. , Fazel, M. , Hlungwani, T. M. , Kahn, K. , Tollman, S. , Cortina‐Borja, M. , & Stein, A. (2013). Childhood Psychological Problems in School Settings in Rural Southern Africa. PLoS ONE, 8(6), e65041. 10.1371/journal.pone.0065041 23776443 PMC3680478

[papt12374-bib-0006] Crawley, E. , Loades, M. , Feder, G. , Logan, S. , Redwood, S. , & Macleod, J. (2020). Wider collateral damage to children in the UK because of the social distancing measures designed to reduce the impact of COVID‐19 in adults. BMJ Paediatrics Open, 4(1), e000701. 10.1136/bmjpo-2020-000701 32420459 PMC7223269

[papt12374-bib-0007] Daniels, I. (2021). Debate: Is there a true global children and young people mental health crisis? Fact or fiction: A South African perspective. Child and Adolescent Mental Health, 26(3), 276–278. 10.1111/camh.12495 34337854

[papt12374-bib-0008] Das‐Munshi, J. , Lund, C. , Mathews, C. , Clark, C. , Rothon, C. , & Stansfeld, S. (2016). Mental Health Inequalities in Adolescents Growing Up in Post‐Apartheid South Africa: Cross‐Sectional Survey. SHaW Study. PLOS ONE, 11(5), e0154478. 10.1371/journal.pone.0154478 27139456 PMC4854374

[papt12374-bib-0009] Deater‐Deckard, K. , & Panneton, R. (2017). Parental stress and early child development ( K. Deater‐Deckard & R. Panneton (eds.)). Springer International Publishing. 10.1007/978-3-319-55376-4

[papt12374-bib-0010] Demkowicz, O. , Ashworth, E. , O’Neill, A. , Hanley, T. , & Pert, K. (2020). Teenagers’ experience of life in lockdown: briefing .

[papt12374-bib-0011] Etard, J.‐F. , Sow, M. S. , Leroy, S. , Touré, A. , Taverne, B. , Keita, A. K. , Msellati, P. , Magassouba, N. , Baize, S. , Raoul, H. , Izard, S. , Kpamou, C. , March, L. , Savane, I. , Barry, M. , Delaporte, E. , Ayouba, A. , Baize, S. , Bangoura, K. , … Yazdanpanah, Y. (2017). Multidisciplinary assessment of post‐Ebola sequelae in Guinea (Postebogui): An observational cohort study. The Lancet Infectious Diseases, 17(5), 545–552. 10.1016/S1473-3099(16)30516-3 28094208

[papt12374-bib-0012] Flisher, A. J. , Dawes, A. , Kafaar, Z. , Lund, C. , Sorsdahl, K. , Myers, B. , Thom, R. , & Seedat, S. (2012). Child and adolescent mental health in South Africa. Journal of Child & Adolescent Mental Health, 24(2), 149–161. 10.2989/17280583.2012.735505 25860182

[papt12374-bib-0013] Fouché, A. , Fouché, D. F. , & Theron, L. C. (2020). Child protection and resilience in the face of COVID‐19 in South Africa: A rapid review of C‐19 legislation. Child Abuse & Neglect, 110, 104710. 10.1016/j.chiabu.2020.104710 32938531 PMC7473143

[papt12374-bib-0014] Golberstein, E. , Wen, H. , & Miller, B. F. (2020). Coronavirus disease 2019 (COVID‐19) and mental health for children and adolescents. JAMA Pediatrics, 174(9), 819. 10.1001/jamapediatrics.2020.1456 32286618

[papt12374-bib-0015] Hawryluck, L. , Gold, W. L. , Robinson, S. , Pogorski, S. , Galea, S. , & Styra, R. (2004). SARS control and psychological effects of quarantine, Toronto, Canada. Emerging Infectious Diseases, 10(7), 1206–1212. 10.3201/eid1007.030703 15324539 PMC3323345

[papt12374-bib-0016] Holmes, E. A. , O'Connor, R. C. , Perry, V. H. , Tracey, I. , Wessely, S. , Arseneault, L. , Ballard, C. , Christensen, H. , Cohen Silver, R. , Everall, I. , Ford, T. , John, A. , Kabir, T. , King, K. , Madan, I. , Michie, S. , Przybylski, A. K. , Shafran, R. , Sweeney, A. , … Bullmore, E. D. (2020). Multidisciplinary research priorities for the COVID‐19 pandemic: A call for action for mental health science. The Lancet Psychiatry, 7(6), 547–560. 10.1016/S2215-0366(20)30168-1 32304649 PMC7159850

[papt12374-bib-0017] Hugo, M. , Declerck, H. , Fitzpatrick, G. , Severy, N. , Gbabai, O.‐B.‐M. , Decroo, T. , & Van Herp, M. (2015). Post‐traumatic stress reactions in Ebola virus disease survivors in Sierra Leone. Emergency Medicine: Open Access, 05(06). 10.4172/2165-7548.1000285

[papt12374-bib-0018] Jiao, W. Y. , Wang, L. N. , Liu, J. , Fang, S. F. , Jiao, F. Y. , Pettoello‐Mantovani, M. , & Somekh, E. (2020). Behavioral and emotional disorders in children during the COVID‐19 epidemic. The Journal of Pediatrics, 221, 264–266.e1. 10.1016/j.jpeds.2020.03.013 32248989 PMC7127630

[papt12374-bib-0019] Kieling, C. , Baker‐Henningham, H. , Belfer, M. , Conti, G. , Ertem, I. , Omigbodun, O. , Rohde, L. A. , Srinath, S. , Ulkuer, N. , & Rahman, A. (2011). Child and adolescent mental health worldwide: Evidence for action. The Lancet, 378(9801), 1515–1525. 10.1016/S0140-6736(11)60827-1 22008427

[papt12374-bib-0020] Kleintjes, S. , Flisher, A. , Fick, M. , Railoun, A. , Lund, C. , Molteno, C. , & Robertson, B. (2006). The prevalence of mental disorders among children, adolescents and adults in the Western Cape, South Africa. African Journal of Psychiatry, 9(3), 157–160. 10.4314/ajpsy.v9i3.30217

[papt12374-bib-0021] Loades, M. E. , Chatburn, E. , Higson‐Sweeney, N. , Reynolds, S. , Shafran, R. , Brigden, A. , Linney, C. , McManus, M. N. , Borwick, C. , & Crawley, E. (2020). Rapid Systematic Review: The Impact of Social Isolation and Loneliness on the Mental Health of Children and Adolescents in the Context of COVID‐19. Journal of the American Academy of Child & Adolescent Psychiatry, 59(11), 1218–1239.e3. 10.1016/j.jaac.2020.05.009 32504808 PMC7267797

[papt12374-bib-0022] Li, A. M. , Chan, C. H. Y. , & Chan, D. F. Y. (2004). Long‐term sequelae of SARS in children. Paediatric Respiratory Reviews, 5(4), 296–299. 10.1016/j.prrv.2004.07.012 15531253 PMC7106002

[papt12374-bib-0023] Lu, C. , Li, Z. , & Patel, V. (2018). Global child and adolescent mental health: The orphan of development assistance for health. PLoS Medicine, 15(3), e1002524. 10.1371/journal.pmed.1002524 29522518 PMC5844520

[papt12374-bib-0024] Lund, C. , De Silva, M. , Plagerson, S. , Cooper, S. , Chisholm, D. , Das, J. , Knapp, M. , & Patel, V. (2011). Poverty and mental disorders: Breaking the cycle in low‐income and middle‐income countries. The Lancet, 378(9801), 1502–1514. 10.1016/S0140-6736(11)60754-X 22008425

[papt12374-bib-0025] Mkhize, D.Z. (2020). First Case of COVID‐19 Coronavirus Reported in SA .

[papt12374-bib-0026] Moore, S. A. , Faulkner, G. , Rhodes, R. E. , Brussoni, M. , Chulak‐Bozzer, T. , Ferguson, L. J. , Mitra, R. , O’Reilly, N. , Spence, J. C. , Vanderloo, L. M. , & Tremblay, M. S. (2020). Impact of the COVID‐19 virus outbreak on movement and play behaviours of Canadian children and youth: A national survey. International Journal of Behavioral Nutrition and Physical Activity, 17(1), 1–11. 10.1186/s12966-020-00987-8 32631350 PMC7336091

[papt12374-bib-0027] Muris, P. , Schmidt, H. , Engelbrecht, P. , & Perold, M. (2002). DSM‐IV–defined anxiety disorder symptoms in South African children. Journal of the American Academy of Child & Adolescent Psychiatry, 41(11), 1360–1368. 10.1097/00004583-200211000-00018 12410079

[papt12374-bib-0028] Nearchou, F. , Hennessy, E. , Flinn, C. , Niland, R. , & Subramaniam, S. S. (2020). Exploring the impact of covid‐19 on mental health outcomes in children and adolescents: A systematic review. International Journal of Environmental Research and Public Health, 17(22), 1–19. 10.3390/ijerph17228479 PMC7698263 33207689

[papt12374-bib-0029] Orben, A. , Tomova, L. , & Blakemore, S.‐J. (2020). The effects of social deprivation on adolescent development and mental health. The Lancet Child & Adolescent Health, 4(8), 634–640. 10.1016/S2352-4642(20)30186-3 32540024 PMC7292584

[papt12374-bib-0030] Patel, V. , Flisher, A. J. , Hetrick, S. , & McGorry, P. (2007). Mental health of young people: A global public‐health challenge. The Lancet, 369(9569), 1302–1313. 10.1016/S0140-6736(07)60368-7 17434406

[papt12374-bib-0031] Patel, V. , Flisher, A. J. , Nikapota, A. , & Malhotra, S. (2008). Promoting child and adolescent mental health in low and middle income countries. Journal of Child Psychology and Psychiatry and Allied Disciplines, 49(3), 313–334. 10.1111/j.1469-7610.2007.01824.x 18093112

[papt12374-bib-0032] Perold, M. D. (2001). The prevalence of anxiety in a group of 7 to 13 year old learners in the Western Cape. Stellenbosch University.

[papt12374-bib-0033] Pierce, M. , McManus, S. , Jessop, C. , John, A. , Hotopf, M. , Ford, T. , Hatch, S. , Wessely, S. , & Abel, K. M. (2020). Says who? The significance of sampling in mental health surveys during COVID‐19. The Lancet Psychiatry, 7(7), 567–568. 10.1016/S2215-0366(20)30237-6 32502467 PMC7266586

[papt12374-bib-0034] Polanczyk, G. V. , Salum, G. A. , Sugaya, L. S. , Caye, A. , & Rohde, L. A. (2015). Annual research review: A meta‐analysis of the worldwide prevalence of mental disorders in children and adolescents. Journal of Child Psychology and Psychiatry and Allied Disciplines, 56(3), 345–365. 10.1111/jcpp.12381 25649325

[papt12374-bib-0035] Sajid, E. , & Saleem, S. (2020). *COVID‐19* * * *: What does it mean for my family and I* * * *? A journey through the eyes of a child* . https://www.childnet.com/ufiles/COVID‐19‐Study‐Report‐(1).pdf

[papt12374-bib-0036] Silverman, W. K. , & La Greca, A. M. (2002). Children experiencing disasters: Definitions, reactions, and predictors of outcomes. In A. M. La Greca , W. K. Silverman , E. M. Vernberg , & M. C. Roberts (Eds.), Helping children cope with disasters and terrorism (pp. 11–33). American Psychological Association. 10.1037/10454-001

[papt12374-bib-0037] Simba, J. , Sinha, I. , Mburugu, P. , Agweyu, A. , Emadau, C. , Akech, S. , Kithuci, R. , Oyiengo, L. , & English, M. (2020). Is the effect of COVID‐19 on children underestimated in low‐ and middle‐ income countries? Acta Paediatrica, 109(10), 1930–1931. 10.1111/apa.15419 32557761 PMC7323043

[papt12374-bib-0038] Sprang, G. , & Silman, M. (2013). Posttraumatic stress disorder in parents and youth after health‐related disasters. Disaster Medicine and Public Health Preparedness, 7(1), 105–110. 10.1017/dmp.2013.22 24618142

[papt12374-bib-0039] Valent, P. (2000). Disaster syndrome. In G. Fink (Ed.), Encyclopedia of stress (Vol. 1). Academic Press.

